# TOGETHER THROUGH DEMENTIA: MEASURING RELATIONSHIP SATISFACTION FOR VETERANS WITH DEMENTIA AND THEIR SPOUSES

**DOI:** 10.1093/geroni/igz038.3460

**Published:** 2019-11-08

**Authors:** Julia Loup, Phoebe Block, Nathalie Rieder, Kimberly Alexander, Teddy K Bishop, A Lynn Snow, Michelle M Hilgeman

**Affiliations:** 1 University of Alabama, Department of Psychology, Tuscaloosa, Alabama, United States; 2 Indiana University of Pennsylvania, Department of Psychology, Indiana, Pennsylvania, United States; 3 Tuscaloosa VA Medical Center, Tuscaloosa, Alabama, United States

## Abstract

Current and prior relationship quality can have significant impacts on the mental and physical health of individuals with dementia (IWD) and their spousal caregivers (CGs). Yet, marital satisfaction is not often assessed. This poster examines use of the Dyadic Adjustment Scale (DAS), a widely-researched marriage counseling tool, for contextualizing interpersonal factors beyond established measures of quality-of-life (QoL), burden, and depression. Community-dwelling spousal CGs (n = 49) and Veterans with dementia (n = 37) completed self-report measures including dyadic relationship satisfaction/distress, mutuality, QoL, depression, burden, and the IWD’s cognitive status. Descriptive statistics, bivariate correlations, and regressions were performed. Scores on the DAS significantly correlated with CG QoL (.567, p < .001), CG depression (-.525, p < .001), CG burden (-.428, p = .002), IWD’s cognitive status (.355, p = .034), and IWD social engagement (-.424, p = .011). IWD-reported DAS scores were positively correlated with IWD QoL (.381, p = .024), CG QoL (.340, p = .043), and IWD subjective health (.360, p =.031). Regression analysis showed CG DAS (b = .188, β = .464, p = .002) and IWD social engagement (b = -2.806, β = -.37, p = .012) are significantly predictive of CG QoL; F(2, 32) = 15.865, p < .001; R2 = .495. Findings suggest that the DAS provides important relationship quality insights and may improve QoL needs assessments for caregiving support and respite services, including CG willingness to continue in the CG role. Examining the DAS longitudinally could also inform intervention delivery as dementia severity progresses.

